# The pervasive impact of frailty on ovarian cancer care and the role of prehabilitation: Qualitative perspectives of key stakeholders^☆^

**DOI:** 10.1016/j.jgo.2024.102173

**Published:** 2024-12-20

**Authors:** Stephanie Cham, Rachel A. Pozzar, Neil Horowitz, Colleen Feltmate, Ursula A. Matulonis, Jennifer C. Lai, Alexi A. Wright

**Affiliations:** a Division of Gynecologic Oncology, Department of Obstetrics and Gynecology & Reproductive Sciences, University of California San Francisco, San Francisco, CA, United States; b Dana Farber Cancer Institute, Boston, MA, United States; c Harvard Medical School, Boston, MA, United States; d Division of Gynecologic Oncology, Department of Obstetrics, Gynecology and Reproductive Biology, Brigham and Women’s Hospital, Dana-Farber Cancer Institute, Harvard Medical School, Boston, MA, USA; e Division of Gastroenterology and Hepatology, Department of Medicine, University of California San Francisco, San Francisco, CA, USA

**Keywords:** Ovarian cancer, Prehabilitation, Frailty, Surgery, Chemotherapy, Qualitative research

## Abstract

**Introduction:**

We performed a qualitative study to explore key stakeholders’ perspectives about the impact of frailty on ovarian cancer care and evaluate a candidate prehabilitation intervention.

**Materials and Methods:**

We conducted semi-structured interviews with patient-caregiver dyads and multidisciplinary clinicians. Patients were ≥ 50 years of age with a new diagnosis of advanced stage (III/IV) ovarian cancer who received cancer-directed treatment (chemotherapy and/or surgery) during the past year and met criteria as pre-frail or frail using the FRAIL scale. We used a semi-structured interview guide to elicit participants’ views on frailty, nutrition, physical therapy, and a candidate prehabilitation intervention. We used inductive and deductive approaches to code and analyze interviews and identify emergent themes and patterns.

**Results:**

Ten patients and caregivers (five dyads) and 10 providers were interviewed. We identified four themes: (1) frailty screening is essential to prevent over- and under-treatment, but underused; (2) stakeholders preferred a multidisciplinary approach to providing tailored care for frail patients over a candidate prehabilitation intervention; (3) patient, family caregiver, and clinician stakeholders reported multiple barriers to prehabilitation programs, including concerns about selection bias, and (4) frail patients and family members are vulnerable and require more psychosocial support.

**Discussion:**

We identified significant barriers to prehabilitation interventions for frail patients with ovarian cancer; initiatives to increase frailty screening and provide tailored multi-disciplinary approaches may have a greater impact.

## 1. Background

Frailty is defined as a multidimensional state of reduced physiologic reserve that results in decreased adaptive capacity and resiliency and increased vulnerability to stressors [1]. This is due to a decline in multiple physiologic systems that are usually—but not always—age-related, and co-exist with chronic disease and/or disability. [2] Patients with ovarian cancer are at particularly high risk of frailty and poor outcomes due to the chronological age of the population affected, hypoalbuminemia inherent to the disease, advanced stage at presentation, and the complexity of treatment, which includes intensive chemotherapy and debulking surgery. Prior research suggests 20 % of patients with ovarian cancer are frail, although these estimates depend on relative indices instead of validated standard measures [3-6]. Similar to other populations, retrospective studies indicate frail patients with ovarian cancer have increased postoperative complications, decreased chemotherapy tolerance, and higher healthcare utilization, compared with non-frail patients [4,5,7].

While there has been increasing acknowledgement of the ways in which frailty impacts ovarian cancer care, there is little consensus on how to best care for frail patients. Increasingly, studies of multimodal prehabilitation interventions (e.g., those combining exercise, nutrition, and psychological support) have highlighted the potential for this approach to reduce perioperative complications and mortality by improving physical function, independence, and mobility prior to surgery in patients with endometrial and colorectal cancers [8,9].

We aimed to explore key stakeholders’ perspectives about the impact of frailty on ovarian cancer care and evaluate a candidate prehabilitation intervention using qualitative interviews, and to seek their perspectives on the design of potential prehabilitation interventions.

## 2. Methods/Design

### 2.1. Aim, Design, Setting

We conducted semi-structured interviews of 20 key stakeholders, including 10 patients and caregivers (five dyads) and 10 multidisciplinary clinicians at Dana-Farber Cancer Institute and Brigham and Women’s Hospital between May and June 2022. EQUATOR Standards for Reporting Qualitative Research were followed [10]. Sample sizes for this study were derived from the concept of informational power required to complete the narrow study aims based on prior systematic reviews and when thematic saturation was reached [11,12]. Using a purposeful sampling method, we reviewed clinicians schedules to identify patients who were 50 years of age or older, had a diagnosis of stage III/IV ovarian cancer within the past year, received cancer-directed treatment, and had clinical notes mentioning treatment difficulties due to frailty-related conditions (e.g., comorbidities, functional limitations, age). We approached patients via telephone or messaging and screened participants for frailty using the validated FRAIL scale at the time of contact for participation in the study [13]. The FRAIL scale identifies older adults at risk for frailty according to five criteria: Fatigue (most or all of the time), Resistance (difficulty climbing a flight of stairs unassisted), Ambulation (difficulty walking a block unassisted), Illnesses (five or more of major comorbidities), and Loss of weight (5 % or more). A score of 1–2 indicates pre-frail and a score of 3 or more indicates a patient as frail. This tool has been validated in community-dwelling adults to correlate relatively closely with more comprehensive geriatric assessments and has been proposed as a rapid clinical frailty screening tool. If patients denoted a score of 1 or more they were invited to participate in the interview.

Patients provided verbal informed consent and were asked to identify their primary family caregivers who participated jointly in the interview. Patients were participants but not involved in design, conduct, or reporting of this study. Clinicians involved in the management of frail patients with ovarian cancer (gynecologic oncologists, medical oncologists, advanced practice providers, registered dieticians, and physical therapists) were approached for participation by email and provided verbal consent. Interviews were conducted by two study members who had no relationship with the patients (SC and RAP), over HIPAA-compliant Zoom videoconferencing, and were de-identified and transcribed. Participants received $50 in recognition of their time. The study was approved by the Dana-Farber Cancer Institute Institutional Review Board.

Baseline demographic and clinical data were abstracted from the electronic medical record. The semi-structured interview guide examined general themes of frailty, nutrition, physical therapy, and prehabilitation intervention design. The candidate prehabilitation intervention would start during neoadjuvant chemotherapy and included a physical therapy program with strength and cardiovascular exercises (an average of four times per week each component for an average of 20 min) led by a physical therapist, a nutrition assessment and follow up led by a registered dietician, and use of a virtual certified health coach/counselor to both motivate and monitor adherence weekly (full details can be seen in the Supplemental document with the interview guide). The physical therapy program proposed was designed in conjunction with input from both physical therapists and an exercise physiologist; examples of both in-person and remote physical therapy programs were discussed.

Data analysis was performed using a combination of inductive and deductive approaches to code and analyze interviews [14,15]. Two members of the study team (SC and RAP) used line-by-line coding to analyze the transcripts and develop an initial codebook. Members independently coded the remaining transcripts, meeting regularly to systematically ensure code application was consistent, each code was then summarized, and emergent themes and patterns were established. The codebook was revisited to ensure that the themes could be accurately applied to each individual code and within and across participants and that thematic saturation had been reached. To enhance validity, a third member of the study team (AAW) reviewed code summaries and themes. We coded all data using the comment function in Microsoft Word, then used a macro to export comments to Microsoft Excel for data management.

## 3. Results

A total of 10 patients and family caregivers (five dyads) and 10 clinicians were interviewed. Participant demographics are displayed in Table 1. Patients were a median of 62 years old (range: 57–86 years), most had stage IV disease, and most received primary or interval debulking and combination chemotherapy. We identified four main themes described below.

### Theme 1: Frailty is vaguely defined, almost never screened for, and the tension between over- versus under-treatment may stem from being unable to differentiate primary frailty that predates the cancer diagnosis and secondary frailty due to the cancer burden (Table 2).

All participants recognized the term “frailty” but had difficulty articulating the syndrome. Most identified chronological age as the primary risk factor but reported a wide range (i.e., 50 to 90 years old). Providers identified comorbidities (e.g., diabetes, pulmonary emboli, and polypharmacy), functional decline, decreased mobility, and inability to perform activities of daily living as a marker and the burden that this imposed on caregivers. Nutritional deficiencies were discussed by clinicians and several endorsed routine screening. Other components of frailty included cognitive impairment and anxiety, which exacerbated the challenge of providing cancer-directed therapy. Almost all clinicians described using an “eyeball” test and reported a desire for a rapid assessment of frailty.

Almost all patient/family caregiver dyads described a clear functional decline. Some reported improvement with treatment, while others noted progressive impairment due to side effects from chemotherapy. Providers described a tension between “overtreating” vs. “undertreating.” Many suspected that frailty impacted chemotherapy tolerance but cited limited empiric evidence to guide treatment modifications. Patients and family caregivers cited significant variation in side effects from chemotherapy including fatigue, gastrointestinal symptoms, and pain, often cyclical in nature. Most cited improvement in later cycles, although many required changes in their treatment. Surgeons discussed the tradeoffs of performing debulking surgery, citing the challenge of balancing the long-term survival benefit and the short-term risks of increased perioperative morbidity and protracted recovery. Almost all gynecologic and medical oncologists reported prioritizing chemotherapy completion over surgery to achieve disease control.

### Theme 2: Clinicians acknowledged the complex needs that frail patients have, but consistently cited lack of time and expertise to address them and encouraged multidisciplinary approaches to help facilitate care coordination (Table 3).

One gynecologic oncologist also discussed the challenge of organizing care for patients at a tertiary care center in conjunction with community clinicians. All clinicians cited the benefit of a program that would identify high risk frail patients and primarily manage their frailty-related complex needs with navigation systems and multi-disciplinary approaches including social work, physical therapy, and psychosocial services.

### Theme 3: Prehabilitation interventions need to be carefully designed for feasibility and acceptability; major barriers identified included social determinants of health, motivation, and the need for tailored assessments (Table 4).

Barriers to participation in prehabilitation interventions commonly cited by patients and family caregivers included the time commitment, self-motivation, and symptom burden. Similarly, providers expressed concern about a frail patient’s motivation to participate in a prehabilitation program. Social determinants of health were also cited as potential concerns by providers, including financial stability, food insecurity, insurance coverage, transportation, caregiver support, and digital literacy/access. Almost all clinicians cited concerns that prehabilitation programs might have limited reach if co-payments for visits with physical therapists or registered dieticians were required, or insurance did not cover the cost of nutritional supplements (e.g., Ensure). Participants indicated that any program that required in-person participation would be infeasible because of travel and dependence upon caregivers for transportation. Motivators for participation cited by patients and family caregivers included improved symptom burden, cancer control, and a desired for interventions that incorporated social connection (e.g., in group setting or with family caregivers). Clinicians felt patients would be motivated if their oncologists recommended the program and if they experienced improved symptoms and increased agency.

Design considerations included virtual options, timing, and personalization of the program. Many patients and caregivers endorsed a preference for a virtual intervention due to distance from the cancer center and the frequency of visits required, with possible in-person evaluations during days of infusions. They also emphasized difficulty participating the week after chemotherapy due to debilitating side effects. Physical therapists reported that a hybrid format may be feasible and important to reassess patients. Selection bias was highlighted as a major potential barrier to scalability, as patients who are motivated may be more likely to participate, while the patients struggling with functional decline or financial barriers may be unable to access programs. Clinicians described the need to demonstrate value and cost-savings to support the adoption, implementation, and sustainability.

### Theme 4: Both patients and providers consistently emphasized the importance of meeting patients’ psychosocial needs, including support from family caregivers and social networks, mental health providers, and social work (Table 5).

All interviewees acknowledged that family caregivers provide instrumental support for frail patients’ medical needs (e.g., organizing medications, communicating with clinicians) and basic needs (e.g., transportation to visits, nutrition), which was noted to be challenging for older patients who may have fewer support systems. The burden of caregiving was acknowledged by both groups. Patients derived social and emotional support from family, friends, and their community. Patients and caregivers endorsed trust and support from their clinicians as an important component of their care. Clinicians underscored that loneliness and isolation were underrecognized. All participants acknowledged the important role of addressing anxiety and depression as a key component of treatment. Clinicians relied on social workers to provide counseling and emotional support and to offer practical support (e.g., resource specialists to address financial toxicity).

## 4. Discussion

In this qualitative study, we found that frail patients with ovarian cancer have complex physical, functional, and psychosocial needs which caregivers and clinicians have difficulty meeting. Most clinicians reported a desire for routine screening, but emphasized that screening needed to be brief due to time constraints. They also underscored the need for screening to be accompanied by robust programs capable of providing comprehensive, multidisciplinary care for frail patients. Patients, caregivers, and clinicians cited multiple barriers to stand-alone prehabilitation interventions, including serious illness, high symptom burdens, physical dependence, social isolation, and social determinants of health.

Geriatric assessments are recommended by the American Society of Clinical Oncology and the National Comprehensive Cancer Network for all patients aged 65 years and older but are rarely performed [16]. Our finding that providers perceive a lack of time and expertise as barriers to frailty screening and management is consistent with prior research [17]. Data demonstrates only 53 % of oncology providers are aware of geriatric assessment guidelines and only 5 % of surgical oncologists use a validated geriatric assessment prior to surgery [17,18]. This is in part because geriatric assessments are cumbersome and time-intensive; 72 % of oncologists cited time as a major barrier to performing screening [17]. Several randomized controlled trials have demonstrated improved quality of life and reductions in chemotherapy side effects and delays with the use of geriatric-driven assessments accompanied by multidisciplinary management [19-22]. In the general surgery literature, frailty screening initiatives have reduced mortality [23]. Similarly, a multi-disciplinary perioperative integrated service (e.g., anesthesia, geriatrics, social work) improved perioperative outcomes, compared to a historical control cohort, and increased the rate of home discharges among high-risk, older adults [23,24]. Importantly, there are a number of rapid screening frailty tools that can be considered for facile implementation for clinical use when complete geriatric assessments are unavailable. This includes the FRAIL scale as described above, the Edmonton Frail Scale (14 questions of various domains including function, nutrition, cognition), and the Clinical Frailty Scale (scored from 1, very fit, to 9, terminally ill based on activities of daily living, dependence, and life expectancy) [13,25,26].

Prehabilitation has been frequently cited as a potential intervention for frail populations, but as our findings suggest, these studies may be limited by selection bias and design. Prehabilitation has been primarily studied in the preoperative context, and a small number of studies have shown improvements in reducing perioperative complications or perioperative functional capacity (e.g., six minute walk distance) in gastrointestinal cancers [27,28]. Notably, neither of these studies performed frailty screening, and both excluded patients with extensive comorbidities and likely excluded a number of frail patients. One randomized trial enrolling frail patients (defined by Fried’s Frailty Criteria of 2 or more) undergoing colorectal cancer resection failed to show any improvement in outcomes [9]. Generalizability of this study may have been limited by a significant proportion of patients undergoing minimally invasive surgery. One meta-analysis of nine studies indicated prehabilitation in frail patients with cancer undergoing elective surgery significantly reduced postoperative complications and hospital length of stay but emphasized the importance of personalized and supervised programs [29]. A small number of trials have also been performed in the palliative care phase showing potential effects on quality of life, fatigue, and fitness but were also limited by recruitment and adherence rates [30]. Prehabilitation may likely be limited by selection bias and may be most feasible in pre-frail and well-resourced patients.

A small number of studies are underway to examine the role of prehabilitation in ovarian cancer patients undergoing neoadjuvant chemotherapy. Preliminary results from one pilot study examining an in-person exercise and nutrition intervention in newly diagnosed ovarian cancer patients also indicated difficulty with accrual with an approach to consent rate of 25 % [31]. Additionally, none are specifically targeted towards frail patients who would benefit the most and most studies target perioperative outcomes with limited data on longer term outcomes important to older adults [20,32,33]. Similar to one prior qualitative study in ovarian cancer, patients identified that they would be motivated to participate in prehabilitation based on clinician recommendation. [34] While we interviewed a heterogenous group of providers, our data also adds key perspectives of stakeholders that would participate in either delivering and/or recommending these interventions, which we believe is important prior to building any intervention.

Functional recovery is a patient-centered outcome that may guide decision-making in older adults, but there are limited data in patients with gynecologic cancers [35]. Several studies have demonstrated that older adults value independence and quality of life as much as (if not more than) traditional oncology outcomes of survival, and is increasingly understood as an important outcome in both surgical and oncologic care of older adults [36]. The Elderly Women With Ovarian Cancer (EWOC)-1 trial randomized patients based on a prospectively generated “geriatric vulnerability score” to evaluate chemotherapy choice based on their score. While this was the first randomized trial to specifically explore treatment choices in patients with geriatric impairments, overall survival was both the primary predictor of the geriatric vulnerability score and the outcome of the trial, limiting the clinical utility of this trial for incorporating treatment preferences [37]. Only one prospective study has evaluated functional recovery in older adults after major cancer surgery with only 0.3 % (*N* = 3) of the trial population included gynecologic cancers [38].

While most trials that enroll older adults focus on over-treatment, our study demonstrates that gynecologic and medical oncologists also worry about undertreating older patients with frailty due to their cancer burden. Indeed, older adults have been shown to be less likely to initiate or be offered standard of care chemotherapy with an analysis of SEER-Medicare database showing 28.8 % received no chemotherapy and 24.7 % received a partial course. Furthermore, older adults have been shown to be less likely to be offered surgery and less likely to undergo surgery with a gynecologic oncology subspecialist [39,40]. Randomized studies that specifically evaluate improvement in geriatric impairments and quality of life in frail patients as a primary outcomes rather than overall survival may be warranted, and has already been studied in the setting of hematologic malignancies [41].

These are several limitations to our study. Our study was performed at a tertiary care center with predominantly White, non-Hispanic patients and the perspectives of interviewees may be limited by this. Patients were screened for frailty at the time of contact for the study typically after receipt of treatment precluding any ability to differentiate frailty due to cancer symptom burden, treatment adverse effects, or both and may not fully represent willingness to participate prior to treatment initiation. Furthermore, we included a wide array of patients of different chronological ages, treatment, and clinical outcomes and experiences are potentially more heterogenous. Our qualitative interview guide was purposefully open-ended and did not delve into details of potential benefits of prehabilitation which could influence participation and responses to the interview and we did find provider recommendation was a motivator. Caregivers were also interviewed as a dyad with the patient which may limit their perspectives but the goal of this study was not intended to address caregiver distress. However, this study has many strengths. Specifically, this study suggests frailty syndromes in patients with ovarian cancer may be independent of chronological age. It also highlights important barriers and facilitators for prehabilitation interventions in this population and provides a diverse array of patient experiences. Finally, it suggests several gaps in the literature and important areas for future research and also emphasized the importance of prehabilitation programs that include psychosocial support.

Frail patients with ovarian cancer may be a unique population that requires multidisciplinary approaches for frailty screening, navigation, and care to ensure that patients receive optimal care with combined chemotherapy and surgery. Future research is needed to better understand the treatment preferences, trajectories, and outcomes of frail patients with ovarian cancer to better tailor treatments to their goals and to ensure equitable delivery of care. The important work of including perspectives of more diverse patient population and community-based and rural centers will be crucial for implementation of any future interventions. Further work to include older adults and incorporate geriatric assessments into randomized studies is greatly needed. Prehabilitation programs may be limited by selection bias and multidisciplinary approaches to identify frail patients and coordination of tailored multi-disciplinary approaches may have a greater impact.

## 5. Conclusions

In conclusion, our qualitative work provided valuable perspectives from important stakeholders on the care of frail ovarian cancer patients. We found that frailty screening is not routinely performed and clinicians rely on subjective assessments and chronological age to guide high-stakes treatment decision making. Our findings also suggest frail patients with advanced ovarian cancer would benefit from multidisciplinary teams to balance risks of under versus over-treatment and improve outcomes.

## Supplementary Material

Supplemental Material

## Figures and Tables

**Table 1:** Interview participant characteristics.

Patient/Family Caregiver dyads (*N* = 10, 5 dyads)	
Patient demographics (*N* = 5)	
Age (median)	64 years (range 57–86)
Race/Ethnicity	
White, non-Hispanic	5 (100 %)
Income level by ZIP code	
<50,000	0 (0 %)
50,000-75,000	3 (60 %)
75,000-100,000	1 (20 %)
100,000-125,000	0 (0 %)
>125,000	1 (20 %)
Insurance Status	
Private	4 (80 %)
Medicare	1 (20 %)
Stage	
III	1 (20 %)
IV	4 (80 %)
Surgery type	
Primary debulking	2 (40 %)
Interval debulking	1 (20 %)
None	2 (40 %)
Chemotherapy adverse effects	
Dose reduction for neuropathy	2 (40 %)
Dose reduction for cytopenia	4 (80 %)
Cycle omitted or reduced to single agent	3 (60 %)
Status at time of analysis	
Alive	4 (80 %)
Deceased	1 (20 %)
Family caregiver relationship (N = 5)	
Spouse	3 (60 %)
Child/children	2 (40 %)
**Clinicians (N = 10)**	
Gynecologic oncologist	3 (30 %)
Medical oncologist	1 (10 %)
Advanced practice provider, medical oncology	1 (10 %)
Registered dietician	2 (20 %)
Physical therapist	3 (30 %)

**Table 2:** The Challenge of Assessing Frailty: Subthemes and Representative Quotes.

Subtheme	Patient/caregiver quote	Provider quote
Frailty assessment subthemes		
Age	[Frailty] makes me think of an older woman… I don’t want to think of myself as an older woman. But sometimes I feel more frail than others. (Patient 1, *69 years old*)	I’m kind of using older and frail as the same. (Clinician 6, *Advanced practice provider, medical oncology*)
Function, loss of independence	I took a shower today because my nurse was coming… but I laid down first so I [had] the energy to do that. (Patient 5, *57 years old*)	I think that’s the biggest one… independence… people want to [be able to] live alone (Clinician 6, *Advanced practice provider)*
Nutrition and weight loss	I can’t always eat… I can’t even look at it. (Patient 1, *69 years old*)	Frailty that starts with dehydration and then goes into calories and protein is one of the biggest challenges (Clinician 4, *Registered dietician*)
Informal assessment	[Frailty] does not sound like my mother. I read the NCCN guidelines on frailty and thought they were vague, anybody over 65 could be in that category… I know people who are younger who exhibit those symptoms, and people like my mom who don’t. (Caregiver of patient 3, *86 years old*)	We can look at a parameter like an albumin and know that’s not great… are we measuring muscle wasting or day-to-day living? (Clinician 4, *Registered dietician*) Its eyeballing people and making a gut decision. (Clinician 6, *Advanced practice provider*)
Over versus under-treatment subthemes		
Role of treatment choice, functional recovery	I was worried about the side effects of the Taxol, particularly the peripheral neuropathy and chemo brain… [my oncologist] said that it wouldn’t make any difference if I just started on the carboplatin in the final outcome (Patient 3, *86 years old*)	Their survival is oftentimes measured in years… With a frail patient you do want to sometimes be aggressive for the benefit of that extended survival… weighing that to the point where you don’t hurt them is the fine line you walk (Clinician 3, *Gynecologic oncologist*) She’s 92, and we gave her single agent carboplatin, despite data suggesting that maybe we should do carboplatin and paclitaxel (Clinician 5, *Medical oncologist*) You’re thinking about whether debulking surgery is going to help them, you could push them the wrong way and can’t recover to get the thing they need, which is chemotherapy (Clinician 1, *Gynecologic oncologist*)

**Table 3:** Multidisciplinary Approaches to Prehabilitation and Patient Navigation: Subthemes and Representative Quotes.

Subtheme	Patient/caregiver quotes	Provider quotes
Care coordination, lack of time	I was sinking and I would contact my oncology team and wasn’t getting a response. (Patient 1, *69 years old*)	They should be seen every day… our patients have more [physical] therapy needs [and] we can’t see them as often (Clinician 8, *Physical therapist*) These patients have very complex needs and we just don’t have the time to coordinate it all (Clinician 6, *Advanced practice provider*)
Multidisciplinary approach	I talked to the social worker, nutritionist, palliative team, a lot of different people” (Patient 5, *57 years old*)	The best thing would be a complex care program and a respite program (Clinician 6, *Advanced practice provider)* A frailty team well versed in how to get people from point A to point B (Clinician 3, *Gynecologic oncologist*) Emotional, social, nutritional, physical support, all those things are components of older patients getting through this (Clinician 1, *Gynecologic oncologist*)

**Table 4:** Design Considerations for Prehabilitation Interventions: Subthemes and Representative Quotes.

Subtheme	Patient/caregiver quotes	Provider quotes
Barriers subthemes		
Time commitment	Four times a week, it’s kind of a lot. (Patient 2, *63 years old*) I had a complication and was in the hospital for a week. I just feel like I would not want to be doing any of this right now. (Patient 1, *69 years old*)	It really needs to be how long it takes muscles to strengthen and show increase in their strength. (Clinician 8, *Physical therapist*)
Cancer symptom burden	I don’t mind exercising like this, but I have to feel better than I do now. (Patient 1, *69 years old*) Your body just can’t, you’re not strong enough to do anything extra. It’s a vicious cycle. (Patient 5, *57 years old*)	You can give them the ideal diet and they can’t eat it as a function of their cancer … I almost have never found a nutrition consult helpful (Clinician 5, *Medical oncologist*)
Self-motivation	I had a physical therapist coming to the house, but I didn’t really want to do like exercises, calisthenics, repeating things. (Patient 5, *57 years old*)	I fear that some older women are not motivated. That’s been my experience. It’s like telling a diabetic to control their sugars and they don’t. (Clinician 1, *Gynecologic oncologist*)
Motivators subthemes		
Clinician recommendation		A lot of patients trust their doctors… getting buy-in from them. (Clinician 8, *Physical therapist*)
Improved cancer or symptom control	Yeah, if it would make my cancer go away. (Patient 5, *57 years old*)	Because you’re going to feel better, motivate them to get better from their disease (Clinician 3, *Gynecologic oncologist*)
Patient engagement	Getting stronger (Patient 2, *63 years old*)	Having some control and input in their in their health… something that they can be engaged in (Clinician 6, *Advanced practice provider*)
Design considerations subthemes		
Virtual versus in-person	An [in-person] program would force her to do things (Caregiver of patient 3, *86 years old*) If you don’t turn it on, it’s not gonna happen (Patient 1, *69 years old*)	In person you’re able to challenge them more than you would at home in terms of exercises (Clinician 8, *Physical therapist*)
Timing of intervention	I I had one week down and two weeks where I was cross country skiing every day. (Patient 4, *62 years old*) There one hour between that time they mix the medicine, and if she’s alert while she’s getting her infusion up to six hours (Caregiver of patient 3, *86 years old*)	Once a month reassessment to progress or regress any exercises (Clinician 8, *Physical therapist*)
Personalization	They’d have to be per individual. (Patient 1, *69 years-old*)	Assessing [and] scaling back and adjusting as needed… we personalize everything (Clinician 8, *Physical therapist*)

**Table 5:** The Importance of Psychosocial Support: Subthemes and Representative Quotes.

Subtheme	Patient/caregiver quotes	Provider quotes
Caregiver support and burden	My husband’s great. He makes me power shakes, because I have to eat something… without his support, I’d be up the creek. (Patient 1, *69 years old*) I’ve moved into the house to help with her care. (Caregiver of Patient 5, *57 years old*)	In [your] 50s or 60s, you might have a spouse who’s more viable [or] children that are younger and around… there seems to be more of a support system [compared to] their 70s or 80s. (Clinician 7, *Registered dietician*) It’s been incredibly stressful for her caregivers. (Clinician 5, *Medical oncologist*)
Psychosocial needs	[She] was talking with somebody for mental health, that was a good step. (Caregiver of Patient 2, *63 years old*)	She was sick and frail and trying to convince them there is a light at the end of the tunnel (Clinician 3, *Gynecologic oncologist*)
Social support	If my friend didn’t offer the place to stay, I don’t know what we would have done. (Caregiver of Patient 1, *69 years old*) [Through] the National Ovarian Cancer Coalition I became good friends with someone who’s going through chemo and I share with her the things that got me through. (Patient 4, *62 years old*)	She is by herself [but] well supported by members of her church and her community. (Clinician 4, *Registered dietician*) I think elderly patients tend to be much more isolated than we recognize. (Clinician 1, *Gynecologic oncologist*)

## Appendix A. Supplementary Data

Supplementary data to this article can be found online at https://doi.org/10.1016/j.jgo.2024.102173.
