# Supplementary material for: The pervasive impact of frailty on ovarian cancer care and the role of prehabilitation: Qualitative perspectives of key stakeholders
Source: J Geriatr Oncol. Author manuscript; Available in PMC 2025 Sep 27. (PMC12476166; doi:10.1016/j.jgo.2024.102173)
Supplement: Supplemental Material [file NIHMS2108040-supplement-Supplemental_Material.docx]

**Appendix A. Qualitative interview guide**

**Part 1 - Multi-disciplinary provider team interview guide**

**Introduction**

*Thank you for agreeing to take part in this interview. My name is Dr. Stephanie Cham and I am in my final year of gynecologic oncology fellowship. I am working with one of our research specialists Dr. Rachel Pozzar who specializes in cancer care quality and patient communication to perform and analyze these interviews. The purpose of today’s interview is twofold. The first is to understand how, from your perspective as a provider, frailty plays a role in caring for ovarian cancer patients. The second is to garner feedback on a prehabilitation intervention we have drafted for frail patients to help support them through their care to ensure it is feasible and acceptable from your perspective as a providers who would be participate and be crucial to this program.*

*We have provided you a copy and reviewed the verbal consent form, please let me know if there are any questions prior to starting. Just as a review, everything you say will be confidential. Information discussed is going to be analyzed as a whole and your name will not be used in any analysis of the interview. Feel free to speak openly and honestly. I will be audio recording the interview so that our team can analyze comments from this interview. At any time if you do not wish to continue or have questions please let me know.*

| Theme | Questions |
| --- | --- |
| Frailty | *We will first start with discussing frail patients with ovarian cancer patients in your practice in general.*   - Can you think back on a recent patient you took care of who is frail – tell me about your experience. - How did you determine the patient was frail? - Did it change your management of the patient? If so, how? - What were your specific concerns while taking care of this patient? - What barriers did you encounter to caring for this patient? - In an ideal world, what resources would be available to help support patients who are frail? |
| General intervention design | *Shown here is the study schema (****Figure 1****). Patients with a new diagnosis would initially be screened by performing a self-administered questionnaire called the FRAIL score. If they score as pre-frail on this questionnaire they will be approached and consented for enrollment into this study.*  *The prehabilitation intervention (****Figure 2****) is a multi-modal program that involves providers including a physical therapist, nutritionist, and a health coach. Patients are referred to a physical therapist if they answer yes to the “Fatigue” “Resistance” or “Ambulation” questions and receive a home-based personalized physical therapy training program, and would be seen virtually by a physical therapy provider for follow up. Patients are evaluated by a nutritionist if they answer yes to the “Loss of weight” question and will undergo a baseline assessment and would receive personalized recommendations ranging from enteral changes to TPN. A health coach would check in with patients weekly to help review and consolidate all the recommendations from the multi-disciplinary team and ensure adherence to the intervention.*   - Based on the schema shown, do you have any feedback for the study design? - What do you believe would be some motivators for your patients to be enrolled in this program? - What are some barriers you foresee to participation? |
| Gynecologic oncology provider specific probes | - Do you currently discuss exercise or strength training with patients? If yes, when? If no, why not? - Do you currently counsel patients on specific nutritional interventions? If yes, what do you counsel. If not, why not? |
| Physical therapy/PT provider specific probes | - How often do you see frail/elderly patients undergoing intensive cancer treatment? - How frequently should physical therapists be involved in the care of these patients? - Which patients should always be seen by physical therapists? - What would make it easier for you to see these patients? Are there specific barriers? - What types of physical therapy and respiratory therapy exercises are helpful for this patient population? - How long and how frequently should patients perform exercises for them to be effective? - How do you personalize exercises to a patient? How often do you change or modify them? - To minimize burden on patients we have constructed this program to be primarily a home-based exercise program. Do you have thoughts about how to motivate this specific population to adhere to the program? - Can physical therapy be delivered virtually? What are the barriers to this approach? Or, what things might make it possible for patients who have limited transportation to participate in physical therapy? - We have two programs we are considering for patients (**Figure 3**). What are some of the benefits and downsides of each of the programs that you foresee? If you had to pick one program from the perspective of a physical therapist is there one you think would be better for patients? |
| Nutrition/RD provider specific probes | - How often do you see frail/elderly patients undergoing intensive cancer treatment? - How frequently should nutritionists be involved in the care of these patients? - Which patients should always be seen by nutritionists? - What would make it easier for you to see these patients? Are there specific barriers? - How do you evaluate frail patients for nutritional deficiencies? - What types of nutritional supplementation are helpful? - What is the optimal time to evaluate an ovarian cancer patient’s nutritional status to intervene? - Do you have a nutrition pathway – how do you make the decision to recommend TPN versus oral intake? Are there any social or patient or provider factors that influence your recommendations? - How often is your recommendation implemented by the medical team? - Would it be feasible to follow up with patients virtually? |

**Figures to be placed into a Powerpoint for interviews**

**Figure 1. FRAIL screening for study**

**
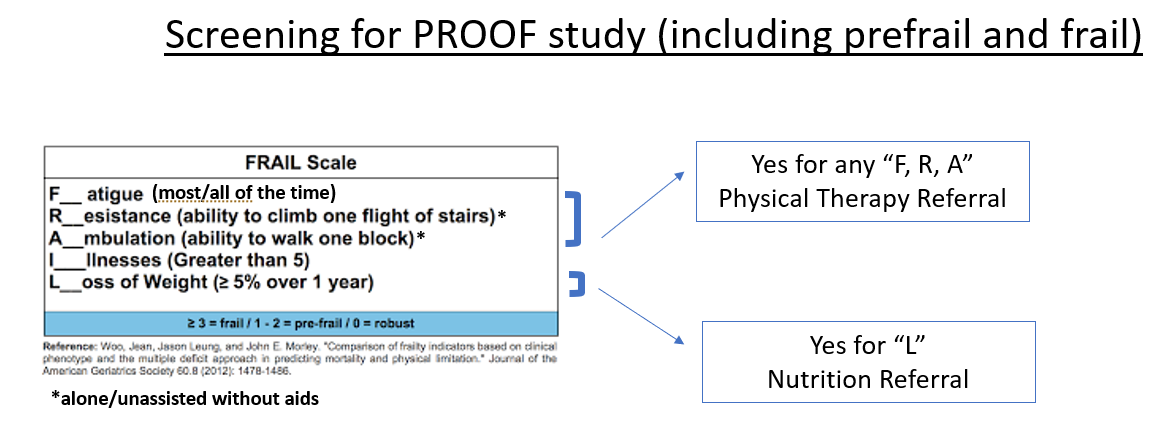
**

**Figure 2. Draft of trial schema**

**
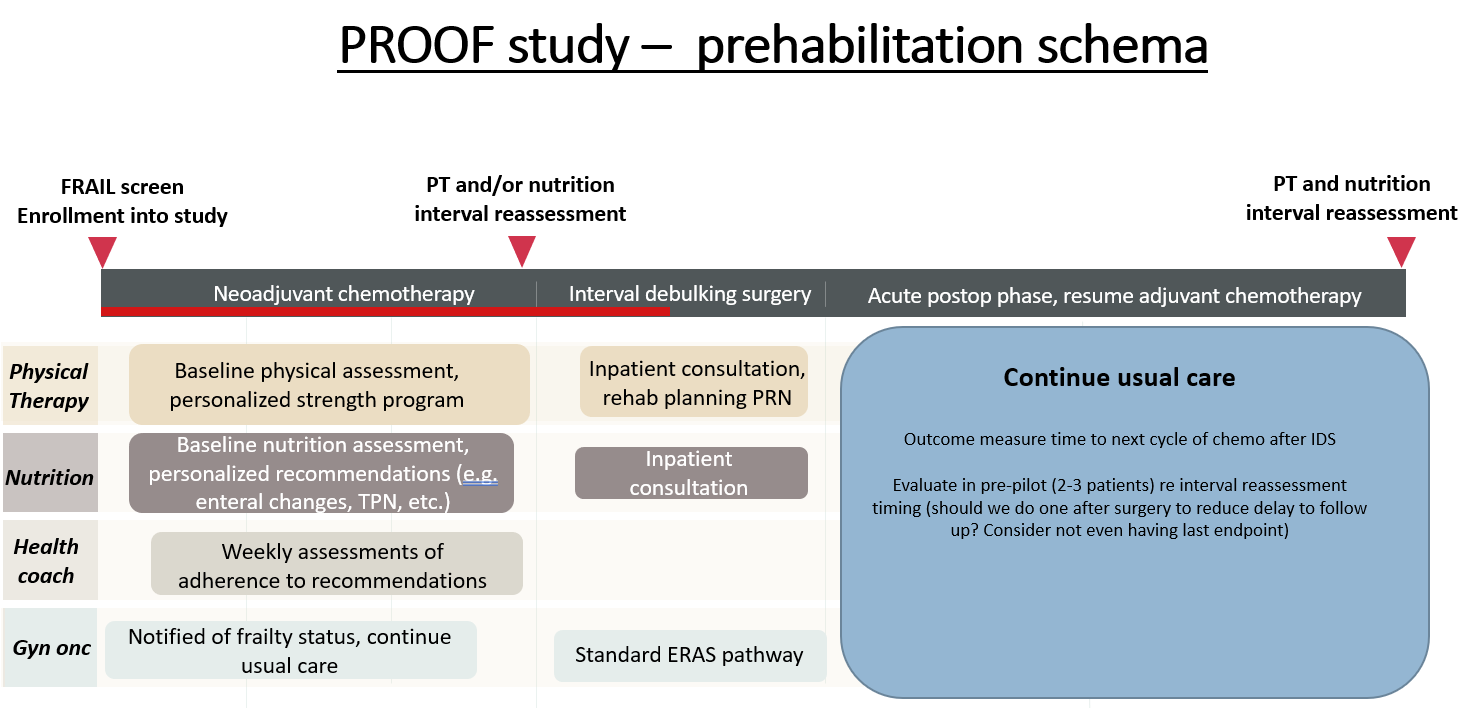
**

**Figure 3. Physical therapy program comparisons**

**Highlighting includes major differences in program

|  | Program 1 (physical therapy led) | Program 2 (exercise physiologist led) |
| --- | --- | --- |
| Visit types | - **1 to 2 visits supervised in person** (Longwood, Chestnut Hill, or Foxborough), additional home based therapy - Must live in the state of MA for insurance reasons - In person visits would be reimbursed | - **4 virtual visits** per week, all home based therapy - Require access to Zoom (e.g. iPad, laptop, cellphone) |
| Strength Training | - 3-5 times a week, mostly by yourself at home, for 30 minutes - Exercises include hip, knee, and balance exercises - Home equipment (strength bands, exercise mat) provided | - 4 times a week all virtually at home supervised, for 30-45 minutes - Exercises include 4 upper body, 4 lower body, and 6 core strengthening exercises - Home equipment (strength bands, exercise mat) provided |
| Cardiovascular Training (e.g. walking, biking, swimming, jogging) | - 10-30 minutes 5x/week - “Moderate” (breathing heavy but can hold short conversation) | - 20 minutes 4x/week - “Moderate to vigorous” (breathing heavy but can hold short conversation or borderline uncomfortable) |

**Part 2 – Patient and caregiver interview guide**

**Introduction**

*Thank you for agreeing to take part in this interview. My name is Dr. Stephanie Cham and I am in my final year of gynecologic oncology fellowship. I am working with one of our research specialists Dr. Rachel Pozzar who specializes in cancer care quality and patient communication to perform and analyze these interviews. The purpose of today’s interview is to discuss how we can help our frail patients through their cancer treatment. You have been invited to take part in this study because based a screening questionnaire has identified you as potentially frail. Frailty is a term used to describe an increased vulnerability to the stress of a new medical problem, such as ovarian cancer. We believe frail patients may benefit from a* *program that helps optimize their health during the treatment of their ovarian cancer. We hope to get your feedback on a program we have designed to help patients like you. It’s really important to us to understand ways to help people like you—and to make sure that we design a program that fits your needs and life in the midst of a cancer diagnosis. Our goal is to help patients like you become stronger—to get better nutrition and strength—so that you feel better during treatment and hopefully have better outcomes. We’re hoping to also get the perspectives of a family member or friend who is close to you so that we can understand their perspective too—this is the reason we invited [family/friend name] to join us.*

*We have provided you a copy and reviewed the verbal consent form, please let me know if there are any questions prior to starting. Just as a review, everything you say will be confidential. Anything we discuss will not be shared with the team of medical professionals taking care of you—and your name will not be included in the interviews when we analyze them. Please feel free to speak openly and honestly. I will be audio recording the interview so that our team can analyze comments from this interview. If you need to stop—or take a break—for any reason, please let me know. We expect this interview should take 30 to 60 minutes.*

| Theme | Questions |
| --- | --- |
| Frailty | *We will first start with asking you both questions about [insert subject’s name here] health and your new diagnosis.*   - When we did an assessment of you, we found that you met criteria for “frailty.” Does the word frailty sound familiar to you? What do you think it means? - Sometimes cancer treatment is challenging. What concerns do you have about what has been recommended to you? Are there specific barriers to completing your treatment (e.g. rides, eating, strength)? - Have you and your [family member/friend] thought about specific strategies to make treatment easier for you? - Other than seeing your oncologist, is there any other type of provider or support you would think could help you during your cancer treatment care? |
| Nutrition | *The next set of questions are specific questions regarding your nutrition.*   - What are some barriers to you currently for having a good diet? - Would you be willing to support your diet by adding supplements, such as protein shakes/drinks or vitamins, to your diet? What are some barriers you foresee? - If a nutritionist thought it would be beneficial to start an intravenous form of nutrition would you be willing to do this? What are some barriers you foresee? - Would you be willing to have regular follow up visits with a nutritionist? Would you prefer for these visits to be in person or would you consider a virtual visit on the computer? |
| Physical therapy | *The next set of questions are specific questions regarding a strength training program*   - How often do you do exercise right now? (e.g. numbers of days per week and time) - What are some of the barriers to exercising right now? - Are there any types of exercise you enjoy? - What types of exercise do you think you could do? (e.g. weights, strength bands, walking, jogging, cycling). - Would you be willing to attend a virtual weekly exercise class? Or a regular virtual visit with a physical therapist? - What would motivate you to exercise? - We have two physical therapy programs patients could possibly participate in (Figure 3). What are some benefits and barriers you for each of these programs. If you had to choose which program you would be willing and able to participate in more and why? |
| Health coach | *This program involves a number of specialists who could help support you throughout your cancer treatment. We recognize that you may have a number of medical appointments during this time and additional appointments or visits may be difficult. The goal of our program is to make sure it is effective without being overly burdensome*   - What are some ways that we could make it easier for you to participate in the prehabilitation program? Are there parts of it that you like? Parts that are less appealing? |
